# Graphical and statistical analyses of the oculocardiac reflex during a non-invasive intracranial pressure measurement

**DOI:** 10.1371/journal.pone.0196155

**Published:** 2018-04-19

**Authors:** Yasin Hamarat, Laimonas Bartusis, Mantas Deimantavicius, Lina Siaudvytyte, Ingrida Januleviciene, Arminas Ragauskas, Eric M. Bershad, Javier Fandino, Jenny Kienzler, Elke Remonda, Vaidas Matijosaitis, Daiva Rastenyte, Kestutis Petrikonis, Kristina Berskiene, Rolandas Zakelis

**Affiliations:** 1 Health Telematics Science Institute, Kaunas University of Technology, Kaunas, Lithuania; 2 Eye Clinic, Lithuanian University of Health Sciences, Kaunas, Lithuania; 3 Department of Neurology, Baylor College of Medicine, Houston, Texas, United States of America; 4 Department of Neurosurgery, Kantonsspital Aarau, Aarau, Switzerland; 5 Department of Neurology, Lithuanian University of Health Sciences, Kaunas, Lithuania; 6 Sports Institute, Lithuanian University of Health Sciences, Kaunas, Lithuania; Oregon Health and Science University, UNITED STATES

## Abstract

**Purpose:**

This study aimed to examine the incidence of the oculocardiac reflex during a non-invasive intracranial pressure measurement when gradual external pressure was applied to the orbital tissues and eye.

**Methods:**

Patients (n = 101) and healthy volunteers (n = 56) aged 20–75 years who underwent a non-invasive intracranial pressure measurement were included in this retrospective oculocardiac reflex analysis. Prespecified thresholds greater than a 10% or 20% decrease in the heart rate from baseline were used to determine the incidence of the oculocardiac reflex.

**Results:**

None of the subjects had a greater than 20% decrease in heart rate from baseline. Four subjects had a greater than 10% decrease in heart rate from baseline, representing 0.9% of the total pressure steps. Three of these subjects were healthy volunteers, and one was a glaucoma patient.

**Conclusion:**

The incidence of the oculocardiac reflex during a non-invasive intracranial pressure measurement procedure was very low and not associated with any clinically relevant effects.

## Introduction

The oculocardiac reflex (OCR), also known as the Aschner reflex, is a cardiac phenomenon that is triggered by physical stimulation of the eye [1]. This phenomenon was first described in 1908 independently by Aschner and Dagnini. Presently, the OCR is considered to be a subtype of a well-known brainstem reflex, the trigeminocardiac reflex [2]. The physiological response of the heart caused by the OCR is characterized by bradycardia or arrhythmia, which may even lead to cardiac arrest [1,3,4]. The OCR can be evoked by pressure on the ocular globe, traction on the extrinsic muscles of the eye, intraorbital injections, hematomas, acute glaucoma, and/or stretching of the eyelid muscles [5]. The stimulus signal is primarily conducted along the ophthalmic division of the trigeminal nerve (V1), which then connects to the ciliary ganglion and visceral motor nucleus of the vagus nerve. The vagus nerve, which is the longest cranial and major parasympathetic nerve, supplies parasympathetic fibers to all major organs, including the heart [6]. The criteria for the evoked OCR is a threshold of decrease in the mean heart rate (MHR) relative to the baseline heart rate (BHR) recorded before physical stimulation of the eye. Various studies have defined thresholds for the evoked OCR [7]. Vrabec et al. (1987) [8] and Eustis et al. (1992) [9] defined a 10% decrease, while others have used a 20% decrease in BHR to define the evoked OCR [10–12]. Finally, Yu & Wang (1991) defined the threshold for the evoked OCR as a decrease of 10 beats per minute compared with baseline [13].

A non-invasive measurement of the absolute value of the intracranial pressure (ICP) has been developed in the Health Telematics Science Institute of Kaunas University of Technology in Lithuania [14]. This method is based on the principles of a non-invasive arterial blood pressure measurement and uses transcranial Doppler (TCD) ultrasonography to assess pulse waves of the ophthalmic artery (OA) during a gradual externally applied pressure (Pe) over a closed eyelid that is transmitted to the eye and orbital (peri-ocular) tissues. The accuracy, precision, and diagnostic reliability of this non-invasive ICP measurement technique have previously been reported [15–17]; however, the incidence of the evoked OCR, which may have important clinical consequences, was not reported.

The aim of this retrospective study was to analyze the presence of the evoked OCR during the non-invasive measurement of ICP when step-wise increases in Pe are applied to the orbital tissues, including the eye.

## Materials and methods

### Equipment and measurement technique

Vittamed 205 (Kaunas, Lithuania), which contains a two-depth TCD with a 2-MHz ultrasonic transducer, was used to collect TCD data during non-invasive ICP measurements. Three versions of the device (Vittamed 205) have been clinically validated since 2009.

**Fig 1:**
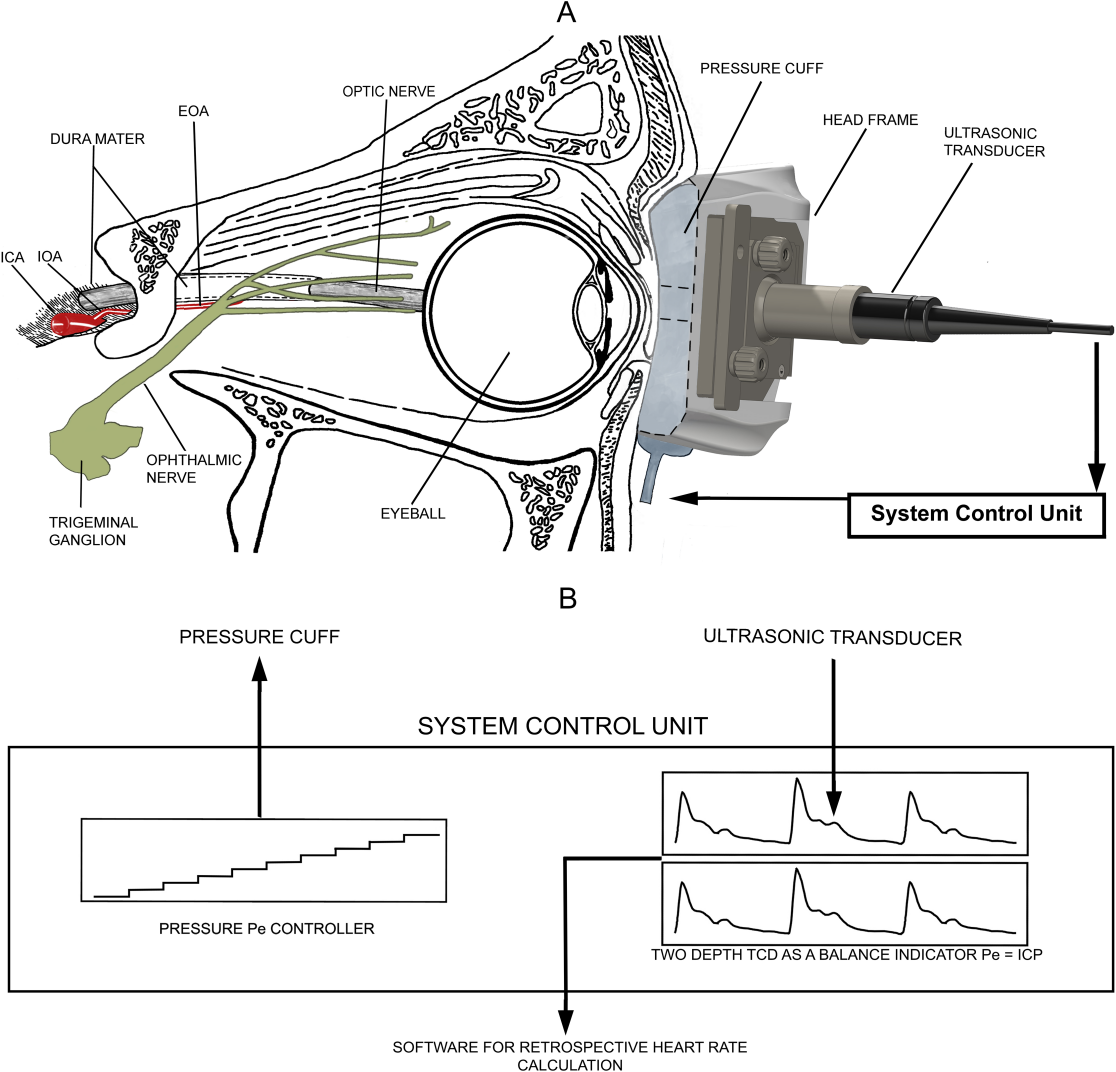
Schematic representation of the non-invasive intracranial pressure (ICP) measurement equipment Vittamed 205. (A) Relevant orbit and brain anatomy in contact with the ICP measurement device. (B) Block diagram of the system control unit. ICA—internal carotid artery; IOA—intracranial part of the ophthalmic artery; EOA—extracranial part of the ophthalmic artery; TCD—transcranial Doppler; Pe—external pressure applied to the ocular globe.

The non-invasive ICP absolute value measurement method uses the intracranial and extracranial parts of the OA [18,19] as a scale to detect the pressure balance between ICP and the gradual externally applied pressure [Pe(t)]. A schematic representation of this non-invasive measurement technique is depicted in Fig 1. Air fills a toroidal-shaped soft plastic cuff installed into the head frame together with an ultrasonic transducer to transmit pressure to orbital tissues. The Pe transmits to the non-compressible orbital tissues and thus, exerts a transmural force on the extracranial OA, but not the intracranial OA, due to segmentation by the dura mater (Fig 1A). A pressure controller automatically increases Pe from 0 to 48 mmHg (maximum) with pressure steps selected by the operator (Fig 1B). The flow velocity pulsations of the OA are continuously monitored by two-depth TCD in the intracranial and extracranial parts of the OA.

### Subjects and settings

Neurological and ophthalmological (glaucoma) patients, as well as healthy volunteers, underwent a non-invasive ICP measurement in the setting of several prospective research studies from October 2009 to December 2016 at the Texas Medical Center at Baylor College of Medicine affiliate hospitals (Baylor Saint Luke’s Medical Center, or Baylor Clinics), Department of Neurosurgery at the Kantonsspital Aarau, Department of Neurology and Eye Clinic at the Hospital of the Lithuanian University of Health Sciences, and the Health Telematics Science Institute of Kaunas University of Technology. The local ethics committee of the Baylor College of Medicine Institutional Review Board, Swissmedic Ethics Committee, and Kaunas Regional Biomedical Research Ethics Committee approved the protocols of the prospective studies whose data were used for this retrospective OCR analysis. All participants provided written informed consent during the prospective studies according to the Declaration of Helsinki.

The inclusion criteria were as follows: all prospective research studies were conducted using non-invasive ICP measurement technology. The exclusion criteria were as follows: (1) measurements that did not contain a BHR—i.e., a heart rate at Pe = 0 mmHg—or (2) the signals of pulse waves of blood flow in the OA collected by TCD were too weak for a reliable heart rate (HR) calculation at any gradual externally applied pressure step. Repeated measurements from the same subject were also excluded.

The clinical charts of all patients included in this study were retrospectively reviewed. The patient demographic information collected included gender, age, and clinical condition. Information concerning the healthy volunteers included gender and age.

### Data processing

**Fig 2:**
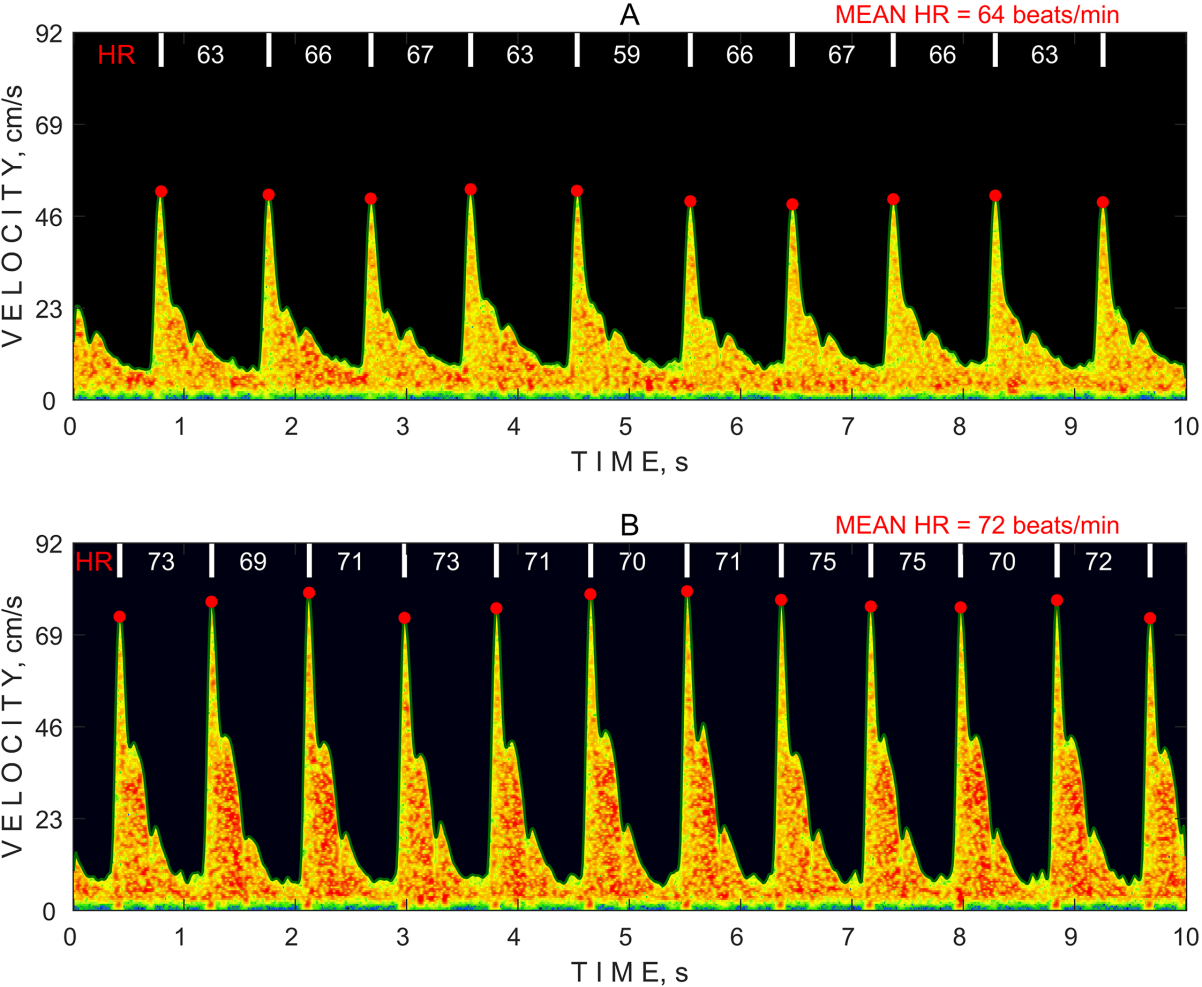
Example spectrograms of pulse waves of blood flow in the ophthalmic artery collected by a transcranial Doppler using different external pressure steps. (A) 0 mmHg; (B) 48 mmHg. HR—heart rate.

An algorithm for an automatic HR calculation was developed to retrospectively test HR variations using TCD data collected during the non-invasive ICP measurements because Vittamed 205 did not have an electrocardiogram (ECG) monitoring channel. Although ECG signals are usually used to examine the OCR, pulse waves measured in the intracranial vessels using TCD remain an accurate technique. Fig 2 illustrates a 10-sec time period of pulse wave spectrograms recorded at different pressure steps during the non-invasive ICP measurement. First, the algorithm calculates a maximum frequency envelope that is plotted as an outer green line of the spectrogram. Second, systolic blood flow velocities and systolic time moments are detected as red dots at every heart beat using a calculated maximum frequency envelope. Finally, the HR is calculated using detected time moments to determine the MHR at each different pressure step of the non-invasive ICP measurement. The HR values and mean HR are shown at different Pe steps in Fig 2A and 2B.

### Statistical analysis

The calculated MHR at Pe = 0 mmHg (minimal physical stimulation of the eye when the ultrasonic transducer is gently resting on the upper eyelid) serves as the BHR value. The differences between the BHR and MHR at every Pe step were calculated and expressed as percentages using the following equation:

$${HR}_{diff}=\frac{MHR–BHR}{BHR}100\%$$ (1)

where HR_diff_ serves as a parameter for the detection of OCR.

Subjects from all groups were enrolled in a repeated-measures analysis of variance to compare HR at different levels of pressure. Kolmogorov–Smirnov (n>50) and Shapiro–Wilk (n<50) tests were used to examine the data distribution normality. Mauchly’s test was used to examine sphericity. A histogram of HR_diff_ values from subjects from all groups was drawn to summarize the HR_diff_ values calculated at all of the Pe steps applied on the ocular globe.

Statistical analysis was performed using IBM SPSS Statistics software (version 23.0; IBM Corporation, Armonk, NY, USA). The level of significance was defined as p<0.05.

## Results

One hundred fifty-seven subjects were included in the OCR analysis. Repeated measurements on the same subject were excluded to eliminate the correlated data points. The mean age (±SD) was 47.6 (±14.4) years (range: 20–75 years), and the male:female ratio was 57:100. The demographic data of the subjects divided into four groups are presented in Table 1.

**Table 1:** Demographic data of the included subjects in this retrospective study.

Subject group	No. of subjects	Age mean±SD (range), years	Gender, female, %
TBI patients	8	53.3±10.2 (37–69)	50.0
Neurologic patients	10	41.0±12.3 (21–60)	60.0
Glaucoma patients	83	53.4±12.5 (24–75)	72.3
Healthy volunteers	56	39.4±13.2 (20–72)	53.6

TBI, traumatic brain injury; SD, standard deviation.

**Fig 3:**
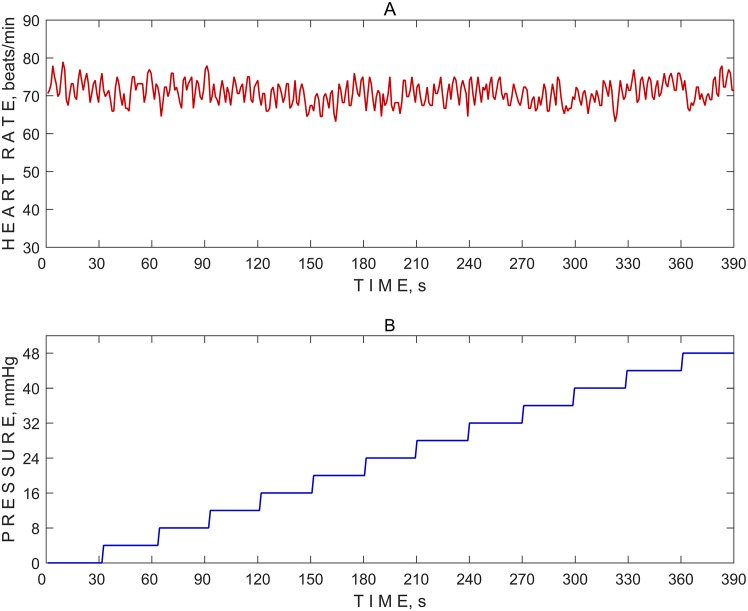
Typical heart rate variation. (A) A heart rate variation in a healthy volunteer. (B) A pressure increase of 4 mmHg per pressure Pe(t) step (time period of approximately 30 sec each) was used on the ocular globe from 0 mmHg to 48 mmHg.

A typical HR variation of a single subject recorded during the non-invasive ICP measurement is depicted graphically (Fig 3).

The maximum external pressure of 48 mmHg and 4-mmHg pressure increase per pressure step are not standard setups for non-invasive ICP measurements. Therefore, the setup can be changed according to the clinical study protocol.

The number of performed measurements using different protocol parameters are presented according to the subject type in all groups in Table 2. The transition time of the pressure increase between two successive pressure steps affects the time period of each pressure step. The time period was approximately 30 sec for each pressure step of the non-invasive ICP measurements; however, a time period of 6 min. per pressure step was used for the 10 healthy volunteers out of 157 subjects according to a different protocol for the non-invasive ICP measurement to determine the number of TCD pulse waves needed per pressure step to reliably measure ICP.

**Table 2:** Protocol parameters for the non-invasive intracranial pressure measurement according to group.

Subject group	Pressure steps, mmHg	Time period, sec.	Body position	No. of measurements
TBI patients	0; 4; 8; 12; 16; 20	30	HUT 20 deg	7
	0; 4; 8; 12	30	HUT 20 deg	1
Neurologic patients	0; 4; 8; 12; 16; 20; 24	30	LD	6
	0; 4; 12; 16; 20; 24	30	LD	2
	0; 4; 8; 12; 16; 20; 24; 28	30	LD	1
	0; 4; 8; 12; 16; 20; 24; 28; 32; 36	30	LD	1
Glaucoma patients	0; 4; 8; 12; 16; 20	30	Supine	83
Healthy volunteers	0; 4; 8; 12; 16; 20	30	Supine	38
	0; 4; 8; 12; 16; 20; 24; 28; 32; 36; 40; 44; 48	30	HDT 30 deg	3
	0; 4; 8; 12; 16; 20; 24; 28; 32; 36; 40; 44; 48	30	Supine	5
	0; 8; 12; 16; 24; 32; 40; 48	360	Supine	8
	0; 8; 12; 16; 24; 32	360	Supine	1
	0; 4; 8; 12; 16; 24; 32; 40; 48	360	Supine	1

TBI, traumatic brain injury; HUT, head up tilt; HDT, head down tilt; Time period, duration of one pressure step; LD, lateral decubitus.

The MHR was calculated separately for pressure steps of each measurement. Repeated-measures analysis of variance was performed to compare the MHR at different levels of pressure. The MHR data matched the normality assumptions according to the Kolmogorov–Smirnov or Shapiro–Wilk test at each different Pe step (the results are presented in Table 3).

**Table 3:** Results of the heart rate and tests of data normality at pressure steps from 0 mmHg to 48 mmHg.

Pe, mmHg	Mean±SD	95% CI of the mean	Med	Min	Max	K-S test value	df	p value	Skewness (SE)	Kurtosis (SE)
0	64.8±8.9	63.4–66.2	64.3	42.4	89.4	0.047	157	0.200	0.25(0.19)	-0.17(0.38)
4	63.8±8.8	62.4–65.3	63.2	41.9	90.1	0.057	148	0.200	0.49(0.20)	0.15(0.39)
8	64.5±8.8	63.1–65.9	63.9	42.3	89.6	0.054	155	0.200	0.34(0.19)	-0.17(0.39)
12	64.5±8.8	63.1–65.8	63.6	42.6	91.0	0.066	157	0.096	0.40(0.19)	-0.07(0.39)
16	64.6±8.8	63.2–66.0	63.9	41.8	90.0	0.056	156	0.200	0.26(0.19)	0.04(0.39)
20	64.5±9.1	63.0–66.0	63.5	41.4	89.2	0.070	146	0.078	0.40(0.20)	-0.21(0.39)
24	63.6±9.6	59.8–67.3	63.4	40.9	87.8	0.973*	28	0.662	0.11(0.44)	0.56(0.86)
28	66.7±9.5	58.7–74.8	66.6	53.4	86.3	0.936*	10	0.508	0.42(0.68)	-0.96(1.30)
32	66.5±8.1	62.6–70.3	69.6	53.1	80.0	0.913*	19	0.083	-0.43(0.52)	-0.94(1.01)
36	65.3±9.3	58.2–72.4	64.6	54.1	79.8	0.920*	9	0.389	0.19(0.72)	-1.54(0.94)
40	66.0±8.2	61.8–70.2	69.3	51.7	80.5	0.917*	17	0.131	-0.37(0.55)	-0.79(0.93)
44	62.5±9.1	54.9–70.2	59.1	53.6	74.1	0.878*	8	0.051	0.45(0.75)	-2.12(1.48)
48	66.3±8.1	62.1–70.5	67.6	51.9	77.3	0.942*	17	0.340	-0.38(0.55)	-1.09(1.06)

Pe, external pressure applied to the ocular globe; SD, standard deviation; CI, confidence interval; Med, median; K-S test, Kolmogorov-Smirnov test; df, degrees of freedom; SE, standard error.

*Shapiro–Wilk test.

Mauchly’s test indicated that the assumption of sphericity was violated (p = 0.047). Therefore, the degrees of freedom were corrected using Greenhouse Geisser estimates of sphericity (ɛ = 0.23). The results showed that no significant effect on the MHR [F(2.71, 18.95) = 0.644; p = 0.581] at different levels of pressure.

**Fig 4:**
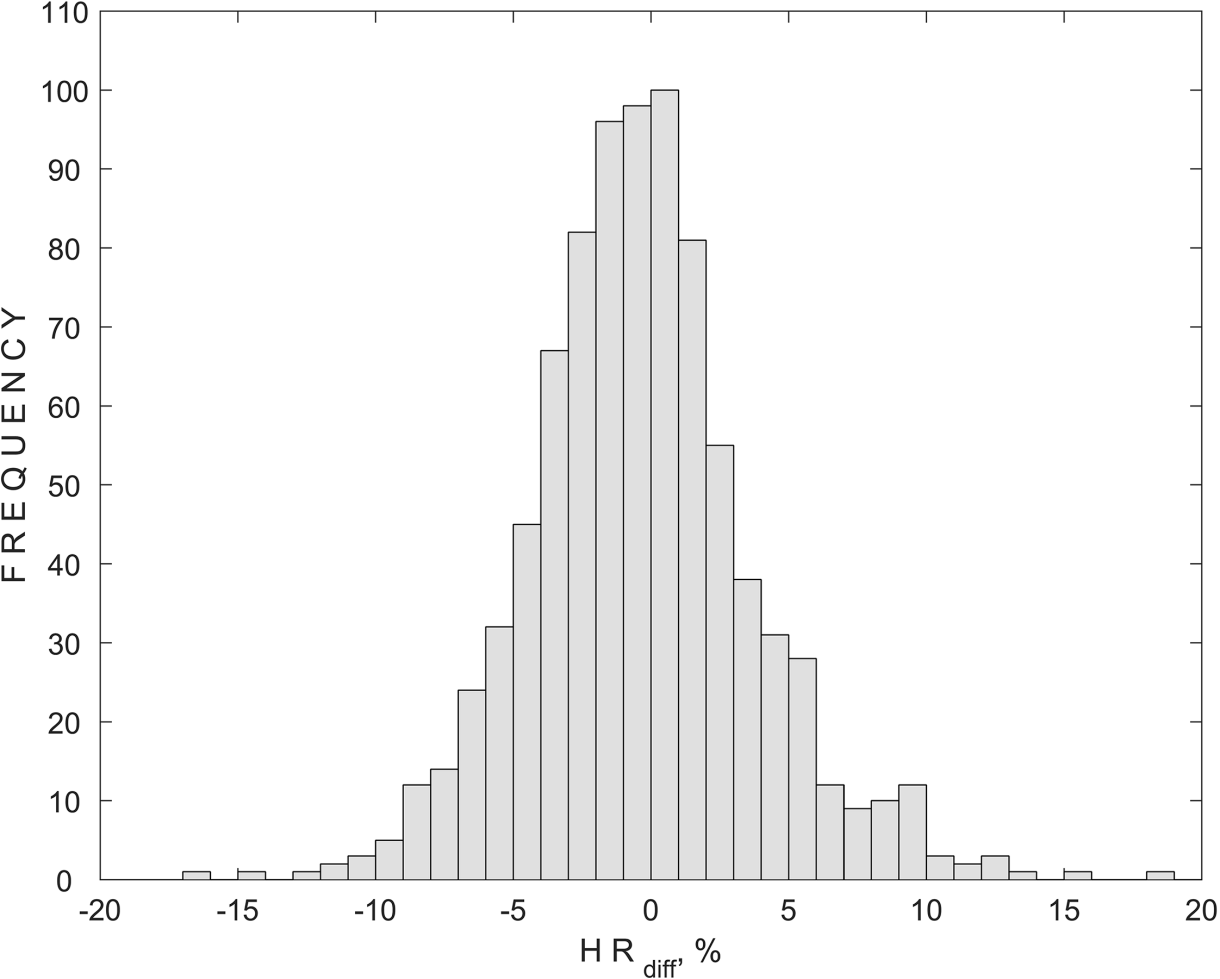
Histogram of HR_diff_ (parameter for the detection of the oculocardiac reflex) for subjects from all groups and 870 external pressure steps applied during all of the non-invasive intracranial pressure measurements.

The differences between the BHR and MHR were calculated and expressed as percentages for the detection of the OCR. A histogram of the HR_diff_ values calculated at every Pe step for subjects from all groups is presented in Fig 4. Obtained HR_diff_ values with a minus sign represent a decrease in the MHR compared with the BHR, while a plus sign represents an increase in the MHR compared with the BHR.

The histogram of HR_diff_ shows a 20% decrease in the MHR compared to the BHR, considering that a firm threshold of the evoked OCR was not reached in any of the 870 pressure steps. A 10% decrease in the MHR, also considered to be a threshold of the evoked OCR, was observed during 8 pressure steps in 4 subjects, representing 0.9% of the total used pressure steps. Among the 4 subjects, 3 were healthy volunteers and one was a glaucoma patient.

## Discussion

Although the OCR was described decades ago, the underlying mechanism has not yet been fully explored [20]. Some studies have focused on understanding the physiological parameters, molecular mechanisms and hemodynamic changes that occur during the OCR [2,21,22]. In this paper, we investigated the prevalence of the OCR using Vittamed 205. In this retrospective study that included healthy individuals and patients with various conditions, a gradual external pressure applied to the ocular globe during the non-invasive ICP measurement did not result in an OCR when using the 20% decrease in the HR criterion and rarely when using the 10% decreased HR criterion.

**Fig 5:**
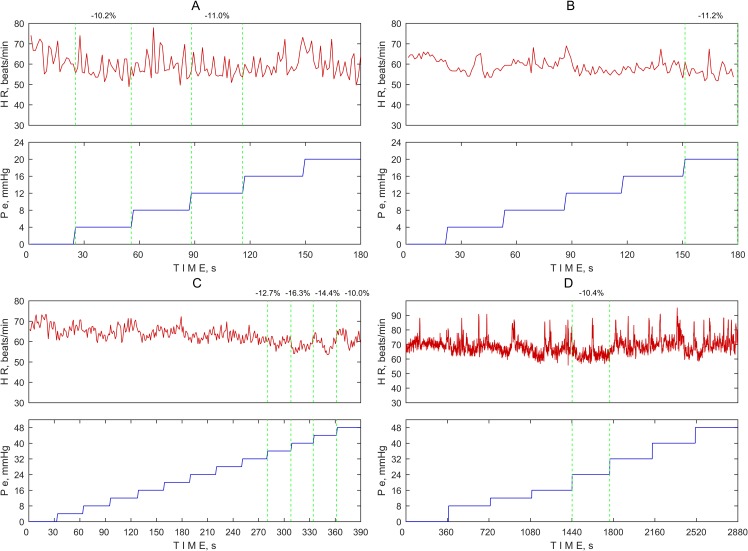
Heart rate (HR) variation at every external pressure step (Pe) applied on the ocular globe in the case of a 10% decrease in the MHR compared to the BHR. (A) Healthy subject. (B) Glaucoma patient. (C) Healthy subject. (D) Healthy subject.

The threshold for defining the evoked OCR remains debatable. Some studies define OCR based on changes in the HR from baseline—a 10% decrease [8,9] or 20% decrease [10–12]. Another study defined a threshold for the evoked OCR as a decrease of 10 beats per minute [13]. A 10% decrease in the MHR compared to the BHR was exceeded at 8 pressure steps (in 3 healthy volunteers and 1 glaucoma patient). The HR variations in these 4 subjects are depicted graphically in Fig 5 together with the gradual pressure steps applied on the ocular globe. It was observed that the HR did not rapidly decrease in association with the increasing external pressure applied on the ocular globe.

The MHR during non-invasive ICP measurements might be influenced by the normal HR variability, and changes in the MHR might not necessarily be related to the OCR. According to Umetani et al. (1998), the HR variability is related to age, and they found that the HR variability ranges from 8.97% (for healthy subjects aged 30 to 49 years) to 13.70% (for healthy subjects aged 80 to 99 years old) [23]. Corrales et al. (2012) showed that, in an active population, the HR variability ranges from 12.96% (for the active men group) to 13.55% (for the active women group) [24]. Nunan et al. (2010) reviewed thirty studies and found that, in healthy adults, the HR variability was 10% [25].

Several important limitations of our study must be mentioned. First, the incidence of the OCR is age dependent. The evoked OCR is more common in children [26]; therefore, most clinical studies of the OCR are reported during strabismus surgery in children [3,5,27,28]. Non-invasive ICP measurements have been conducted only in adult subjects to date; therefore, we cannot assume that a similar procedure could produce the OCR in children. The mean age of all subjects included in this retrospective analysis was 47.6 years (range: 20–75 years). However, this non-invasive ICP measurement device is intended to be used mostly for adult glaucoma patients, not children or anesthetized patients. Next, we did not implement ECG monitoring in our study participants given that clinically significant changes in the HR and/or arrhythmias were not expected in this population; therefore, it is possible that the measurement procedure could have produced non-detectable cardiac arrhythmias that did not transmitting pulse waves to the intracranial vessels that were being measured. However, this is unlikely as a skipped beat or arrhythmia would still be detected as an irregularity of the pulse waves or as an interval between pulse waves as measured in the intracranial vessels.

It is likely that the low magnitude of the tractional force on the extraocular muscles or the level of applied pressure on the ocular globe was the reason for not observing the OCR in our study. Previous studies also have found that to be important factor in producing the OCR. Blanc et al. (1983) and Vrabec et. al. (1987) reported the incidence of the OCR using the force of acute or slow gradual traction and reached a peak at not less than 150 grams (maximum = 300 grams) in extraocular muscles [5,8]. They found that slow gradual traction often failed to evoke the OCR. However, an acute force of traction reaching up to 300 grams evoked the OCR quite often (86.7%). The force of traction using a 4–0 suture silk loop, as described by Blanc et al., (1983) produced a pressure of hundreds of mmHg to evoke the OCR [5]. Therefore, the lack of an evoked OCR in this retrospective analysis may be explained by the slow increase in pressure (4 mmHg pressure increase per pressure step each 30 sec) on the ocular globe and the low level of the maximum applied pressure (48 mmHg).

It is even speculated that OCR responses in humans might not occur in resting humans but only become evident during stress (such as diving or operations) when oxygen requirements are increased [21]. Subjects did not experienced this type of stress during the non-invasive ICP measurement using Vittamed 205.

## Conclusions

In this retrospective study, we observed a very low incidence of the OCR during a non-invasive ICP measurement when the gradual external pressure was increased from 0 mmHg to 48 mmHg on the ocular globe in adult neurological, ophthalmological (glaucoma) patients and healthy volunteers. Further studies are needed to exclude a clinically significant OCR in children or patients with underlying cardiovascular disease.
